# A pragmatic cluster randomised controlled trial of an oral health intervention for people with serious mental illness (three shires early intervention dental trial)

**DOI:** 10.1186/1745-6215-14-S1-P19

**Published:** 2013-11-29

**Authors:** Hannah Jones, Clive Adams, Andrew Clifton, Patrick Callaghan, Peter Liddle, Heather Buchanan, Vishal Aggarwal

**Affiliations:** 1 University of Nottingham, Nottingham, UK; 2 University of Huddersfield, Huddersfield, UK; 3 University of Manchester, Manchester, UK

## 

People with mental illness have poor oral health compared to those without due to medication side effects, issues with self-care, barriers to treatment and poor recognition of dental problems. Guidelines recommend giving oral health advice and monitoring oral health for people with mental illness, but this is not reflected in current practice and Cochrane reviews found no existing randomised trials of these interventions.

The aim is to investigate whether a dental checklist, preceded by dental awareness training for Care Co-ordinators in Early Intervention in Psychosis (EIP) teams, affects oral health and behaviour of people with serious mental illness.

The intervention (dental checklist) was adapted from guidelines with clinicians and service users. The checklist comprises questions regarding current oral health state and practice, and general mental state. EIP teams were randomly allocated to either the intervention or to continue with standard care for 12 months. Both arms of the trial were balanced for team size and location. Intervention team Care Co-ordinators received 30 minutes of dental awareness training before initial use of the checklist with their service users. Twelve months later the checklist is repeated. Control group Care Co-ordinators continue to deliver standard care for 12 months before receiving dental awareness training and using the checklist with service users.

This collaborative study design is unique. The simple intervention and method shows how a bottom-up design may work. These trials are potentially powerful and can produce interventions that, if effective, could be widely implemented with little time and cost implications.

